# Ultrabroadband, More than One Order Absorption Enhancement in Graphene with Plasmonic Light Trapping

**DOI:** 10.1038/srep16998

**Published:** 2015-11-19

**Authors:** Feng Xiong, Jianfa Zhang, Zhihong Zhu, Xiaodong Yuan, Shiqiao Qin

**Affiliations:** 1 College of Optoelectronic Science and Engineering, National University of Defense Technology, Changsha, 410073, China; 2 State Key Laboratory of High Performance Computing, National University of Defense Technology, Changsha, 410073, China

## Abstract

This paper presents an comprehensive study of light trapping and absorption enhancement in graphene through metallic plasmonic structures and shows a strategy to realize both ultrabroadband and strong absorption enhancement. Three different plasmonic absorber designs are investigated by numerical simulations. The excitation of localized plasmons in the metallic structures significantly enhances the interactions between graphene and light at the resonances. By employing a splitted cross design for plasmonic resonant antennas and integrating two types of sub-antennas with different sizes, more than 30% of optical absorption in monolayer graphene is realized in a ultrabroad spectral range from 780 to 1760 nm. This enhancement functionality can be translated to any wavelength band from ultraviolet to terahertz ranges by modifying the geometric design of the plasmonic structure and can be applied for other two dimensional materials and their heterogeneous structures. It may significantly improve the efficiency of optical devices such as broadband photodetectors and solar cells based on graphene and other two-dimensional materials.

In the past few years, graphene and other two-dimensional materials have emerged as promising materials for optics and optoelectronics^1^,^2^,^3^. Graphene, a single layer of carbon atoms arranged in plane with a honey comb lattice, have been studied for a variety of applications such as transparent electrodes^4^,^5^, optical modulators^6^, saturable absorbers and ultrafast lasers^7^,^8^ as well as tunalbe plasmonic devices^9^,^10^,^11^,^12^. Among these applications, graphene photodetectors have attracted significant research efforts^13^. Graphene is gapless which enables carrier generation by light absorption over a very wide energy spectrum. Moreover, graphene shows very high carrier mobility and exhibits a nearly wavelength-independent absorption of about 2.3% in the visible and near infrared range which is related to the fine structure constant^14^,^15^. These properties make graphene a novel material for high speed and broadband photodetectors with a couple of exciting demonstrations having been made recently^16^,^17^. However, even though such an absorption of 2.3% is quite remarkable considering its atomical thickness, it is too weak in absolute terms and severely limit the performance of graphene photodetectors. So lots of research efforts have been made to enhance its interactions with light.

Many approaches have been proposed to enhance the absorption in graphene. One is placing it in an optical cavity, which enables light to pass through the graphene film multiple times. Different cavities have been employed including F-P microcavities^18^,^19^ and two dimensional photonic crystal cavities^20^,^21^. The improvement of absorption by this approach generally comes at the expense of spectral bandwidth. Another approach is the coplanar integration of graphene with an optical waveguide^22^,^23^,^24^. The interaction distances between graphene and light can be long by this method and broadband and high absorption in graphene is possible, but it is not applicable for the detection of free space light unless it is coupled into the waveguide. Besides, improvement of graphene absorption through light trapping by plasmonics have attracted significant attentions. In the mid-infrared and THz ranges, doped graphene itself is a plasmonic material and can support the excitation of graphene plasmons, the intrinsic collective charge oscillations of the 2D electron liquid inside graphene^25^,^26^. Enhancement of optical absorption by graphene plasmonics have been intensively studied^27^,^28^,^29^. Perfect absorption have been theoretically predicted in nanostructured graphene backed by a mirror or in a free standing structured graphene film under the illumination of two coherent beams^30^,^31^. Recently, more than one order of absorption enhancement in arrays of doped graphene disks have been experimentally demonstrated^32^,^33^. At short wavelengthes in the visible and near infrared range, localized plasmons of metallic nanostructures have been exploited for light trapping and absorption enhancement in graphene^34^,^35^,^36^,^37^. Significant improvement in performance has been demonstrated by fabricating metallic plasmonic nanostructures near the contacts of graphene based photodetectors. Particularly, strong resonant enhancement of light absorption in graphene integrated with a metamaterial absorber has recently been proposed^38^. However, realizing both broadband and strong absorption enhancement remains a challenge.

In this paper, we investigate the light absorption of graphene integrated in plasmonic absorbers. Three different structures are studied and a strategy to realize ultrabroadband light trapping and absorption enhancement in graphene is proposed. It is shown that >30% light absorption by monolayer graphene can be realized in a spectral range from 780 to 1760 nm with the proposed design. The enhanced absorption is more than one order higher than that by a freestanding graphene film without enhancement.

## Results and Discussion

**Figure 1:**
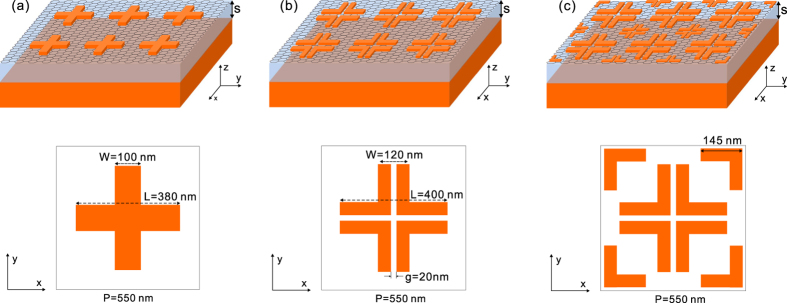
Plasmonic absorbers for light trapping and absorption enhancement in graphene. (**a**) Schematic of a plasmonic absorber comprising arrays of periodical metallic crosses backed by a metallic mirror. (**b**) A similar structure as in (**a**) but the metallic crosses have 20 nm wide cross gaps. (**c**) Schematic of a absorber consisting of two types of crosses with different dimensions to expand the absorption bandwidth. The top views of unit cells with geometric parameters are shown in the bottom.

Figure 1 shows the schematic illustration and geometric parameters of three different structures for plasmonic light trapping and absorption enhancement in graphene. The metallic structures consist of arrays of periodical metallic crosses backed by a metallic mirror, which is a well-known design for plasmonic perfect absorbers^39^. From the top to the bottom of each structure are an array of metallic particles, the monolayer graphene, an insulator layer with a thickness of *s* = 80 *nm* and a metallic reflection mirror, respectively. The graphene film is in direct contact with the metallic crosses. The period is *P* = *Px* = *Py* = 550 *nm*. The insulator layer is assumed to be lossless with a refractive index of 1.5. The metallic crosses and mirror are made of gold. The thickness of the crosses are 30 nm. The incident light excites localized plasmons in the metallic structures, which trap light in the near-field and lead to strong absorption. As the graphene film is now integrated in the structures, a significant proportion of light will be absorbed by the graphene which will far exceed the absorption by a free standing graphene film.

**Figure 2:**
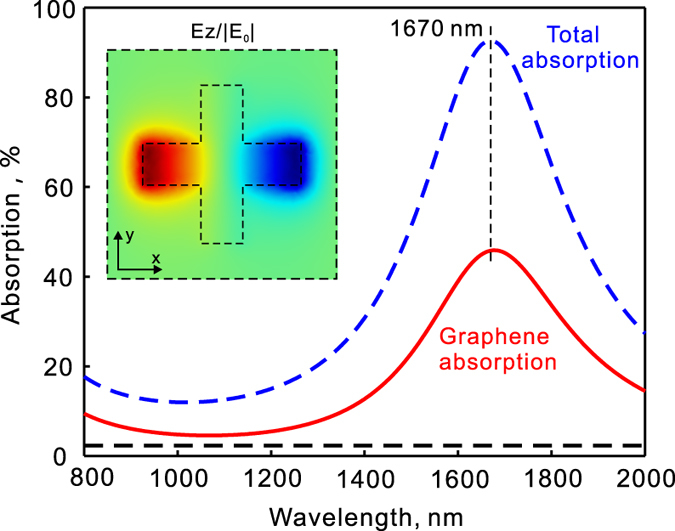
Absorption enhancement in graphene by plasmonic light trapping. (**a**) Simulated spectra of total absorption (blue dashed line) as well as absorption in the graphene (red solid line) for the structure in Fig. 1a. The inset shows the field distributions at the resonance as denoted by the vertical dashed lines. The light is incident at normal direction with its electric field polarized along the x-direction. The field is plotted at the x–y plane that is 25 nm below the bottom surface of the gold crosses. The flat dashed line shows the absorption in a free standing graphene without enhancement.

First, we consider the structure in Fig. 1a. It consists of arrays of ordinary metallic crosses backed by a reflection mirror. This is a typical design for polarization-independent plasmonic perfect absorbers^39^,^40^. Figure 2 shows the numerically simulated absorption spectra under the illumination of a plane wave at normal incidence. Total absorption is shown in dashed line and absorption in the graphene is shown in solid line. It exhibits a single resonance in the studied spectral range. The inset shows the field distributions at the resonance. As is well known, an electric dipole mode is excited in the arm of the cross which forms a magnetic mode along with its image dipole by the reflection mirror. At the resonance wavelength of 1670 nm, the total absorption reaches about 93% while the absorption in the graphene is about 46%. As a reference, the absorption in a free standing monolayer graphene is also shown (the flat dashed black line). The resonant absorption in graphene is enhanced by 20 times with the plasmonic light trapping. The total absorption as well as absorption in graphene decreases as the wavelength of light is away from the resonant wavelength. Here the plasmonic resonance has a quality factor of about 5 and the graphene absorption keeps higher than 30% in a spectral band of about 280 nm (from the wavelength of 1546 nm to 1825 nm).

**Figure 3:**
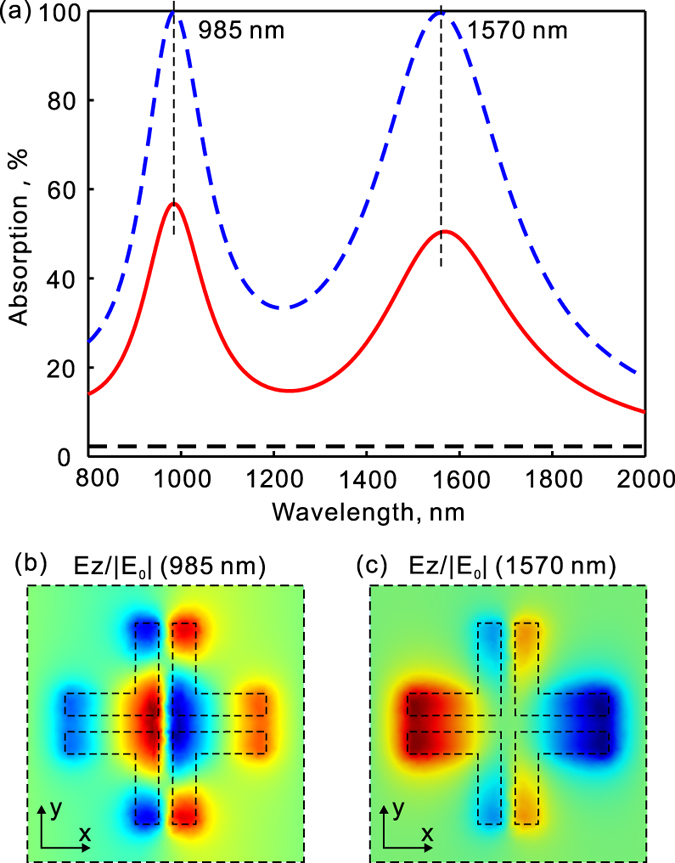
Absorption enhancement in graphene by plasmonic light trapping with dual resonances. (**a**) Simulated spectra of total absorption (blue dashed line) as well as absorption in the graphene (red solid line) for the structure in Fig. 1b. The light is incident at normal direction with its electric field polarized along the x-direction. (**b**,**c**) show the field distributions at the two resonances as denoted in (**a**) by the vertical dashed lines. The field is plotted at the x–y plane that is 25 nm below the bottom surface of the gold crosses. The flat dashed lines show the absorption in a free standing graphene without enhancement.

Figure 3a shows simulated absorption spectra for the structure in Fig. 1b. The structure in Fig. 1b is similar to that in Fig. 1a but the metallic crosses have a 20 nm wide cross gap in the center. It exhibits two resonance peaks in the studied spectral range. At the resonance wavelength of 1560 nm, the total absorption and absorption in the graphene reach about 99.6% and 50%, respectively. The graphene absorption is enhanced by about 22 times at the resonance. The origin of this resonance is similar to the resonance in Fig. 2a as shown by the field distributions (see Fig. 3c and the inset in Fig. 2). Besides, there is an other resonance at about 985 nm with the total absorption and graphene absorption reaching 99.8% and 57%, respectively. This resonance is attributed to the excitation of a higher order mode as shown by the field distribution in Fig. 2b. So the introduction of a cross gap in the metallic cross can lead to dual resonant bands for absorption. Moreover, the middle gap could lead to field enhancements in the graphene plane which is beneficial for absorption enhancement in the graphene film.

**Figure 4:**
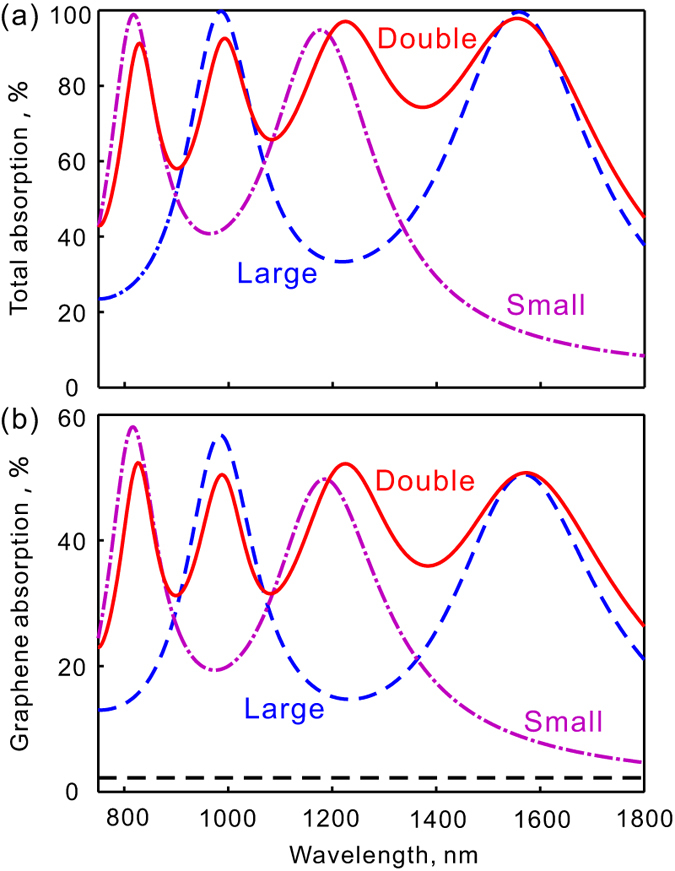
Broadband absorption enhancement in graphene by plasmonic light trapping. The solid red line shows the simulated spectra of absorption in the graphene for the structure in Fig. 1c. The absorption for the structures with just one type of crosses is described by the dashed blue line (large crosses) and dashed purple line (small crosses). The flat dashed line shows the absorption in a free standing graphene without enhancement.

Plasmonic resonators generally have an absorption cross-section bigger than its geometric size and multi-resonances can be realized by integrating two or more sub-resonators with different sizes in one unit cell^39^. Figure 4 shows the absorption spectra for the structure in Fig. 1c (solid red curves) as well as those for its two individual sub-structures (dash-dotted purple and dashed blue curves). The structure contains two crosses with different dimensions in each unit cell. The side width of the large cross is 400 nm (the side width of each L-shaped part is 190 nm and the gap width in the middle is 20 nm) and that of the small cross is 310 nm (the side width of each L-shaped part is 145 nm and the gap width in the middle is 20 nm). The combined structure exhibits four resonances in the studied spectral range including both the resonances of the small cross (dash-dotted purple curve) and resonances of the large cross (dashed blue curve). As a result, light trapping and strong absorption enhancement is now realized in a broadband wavelength range. The total absorption of the whole structure reaches more than 97% at the four resonances and exceeds 58% at range from 770 nm to 1740 nm (see Fig. 4a). At the same time, the light absorption in the monolayer graphene reaches more than 30% in an ultrabroad spectra range from 780 to 1760 nm (see Fig. 4a).

In the above structures, the graphene film is in direct contact with the metallic crosses. This is just the case for many demonstrated graphene photodetectors^34^,^35^,^36^. Metals such as gold will lead to the change of graphene’s Fermi energy and get doped graphene^41^. The modification of the Fermi level is not limited only to graphene underneath the metal but also extends hundreds of nanometers into the uncovered graphene with varied doping levels^42^. Doping will affect the optical properties of graphene, particularly for light with photon energy around and below twice of the graphene’s Fermi energy^15^. According to previous study, the change of Fermi level induced by gold contact is generally less than 0.5 eV^43^. Even though metal-induced doping may lead to some decrease of absorption enhancement at the long wavelength range, broadband enhanced absorption can still be realized at visible and near infrared ranges.

**Figure 5:**
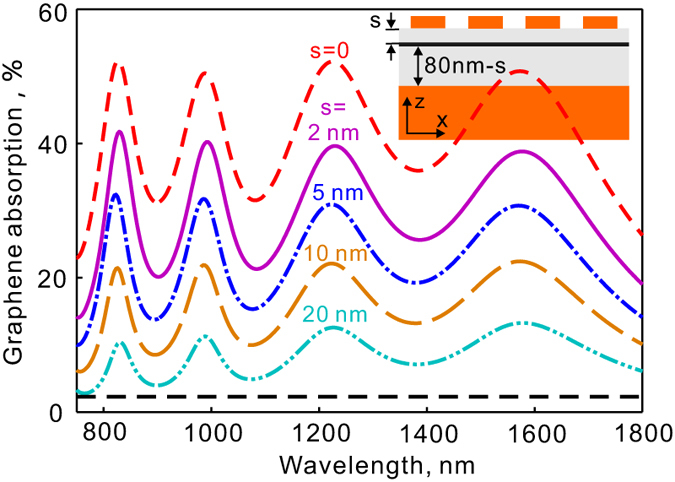
Light absorption in graphene for different separations between the graphene layer and metallic crosses. The separation varies from 0 to 20 nm. The separation layer is assumed to be lossless with a refractive index of 1.5. The flat dashed line shows the absorption in a free standing graphene without enhancement.

Sometimes it may be necessary to have an insulator layer between the graphene film and the metallic plasmonic nanostructures (e.g., in order to protect the graphene film from unwanted doping and contamination). Figure 5 shows the absorption spectra of graphene with different separations between the graphene layer and metallic crosses. The inset in Fig. 5 shows the schematic cross section of the structure. The geometric parameters of the metallic crosses are just the same as in Fig. 1c. As is known, the metallic nanostructure here employs the typical design for plasmonic perfect absorbers and it forms a high impedance surface with the electric field mainly confines near the top surface (metallic crosses) of the structures. Not surprisingly, the absorption in graphene drops with the increase of the separation. As the separation is zero, the absorption in graphene is about 52.3%, 50.5%, 52.2%, 50.8% at the four resonances of 829 nm, 988 nm, 1225 nm, 1573 nm, respectively. As separation is *s* = 2 *nm*, the resonant absorption in graphene decreases to 41.8%, 40.2%, 39.6%, 38.8%, respectively. They drop to 32.4%, 31.7%, 30.9%, 30.7% at the separation of *s* = 5 *nm* and 10.4%, 11.3%, 12.6%, 13.3% at the separation of *s* = 20 *nm*. However, the absorption in graphene can still exceed 13.8% in the spectral range from 780 to 1800 nm when the separation is *s* = 5 *nm*.

**Figure 6:**
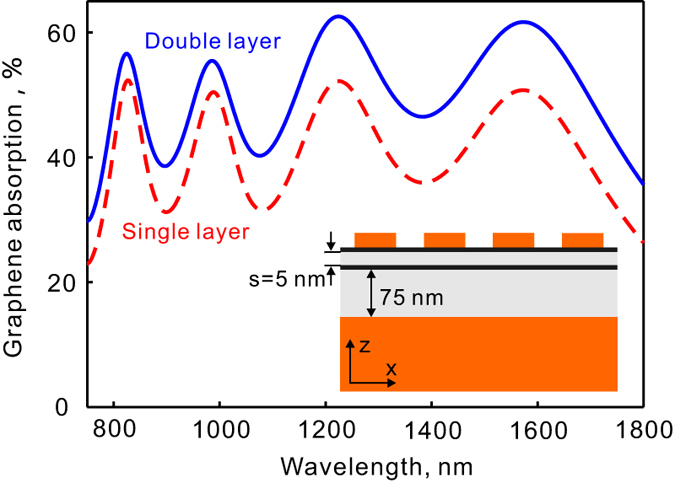
Broadband absorption enhancement in a graphene double layer by plasmonic light trapping. The two graphene monolayers are separated by a thin tunnel barrier with a thickness of 5 nm. The top layer of graphene is in contact with the metallic crosses. For simplicity, the separation layer is assumed to be lossless with a refractive index of 1.5.

Besides monolayer graphene and other two-dimensional materials, heterostructures of two-dimensional materials with vertical stacks have attracted a great deal of attention for their potentials for optoelectronic applications. High-efficient and broadband photodetectors based on graphene double-layer heterostructures have been reported^44^,^45^. They consist of a pair of stacked graphene monolayers separated by a thin tunnel barrier. Our proposed structure can also be used for light trapping and graphene absorption enhancement in this type of devices. Figure 6 shows the absorption in a graphene double-layer heterostructure enhanced by plasmonic light trapping. The maximum absorption in the two graphene layers reaches about 62.6% (about 43.3% in the upper graphene layer and 19.3% in the lower layer) at the resonance of 1225 nm which is about 10% higher compared to the monolayer graphene. In the studied spectral range, the increase of absorption in graphene is slightly higher at longer wavelengths (about 10%) than that at shorter wavelengthes (about 5$ to 6%). This is due to the relatively larger spatial extensions of localized plasmonic fields near the metallic crosses at longer wavelengthes.

**Figure 7:**
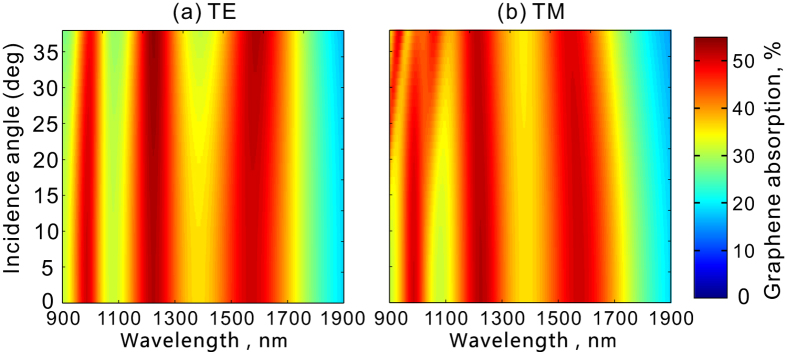
Angle dependence of the resonant absorption in graphene for (a) s-polarized (TE) and (b) p-polarized (TM) light. Here the graphene is in contact with the metallic crosses (*s* = 0) as in Fig. 1c.

Figure 7 shows the angle dependence of absorption. Here the structure is the same as in Fig. 4. In order to avoid diffraction effects, we only consider wavelengthes from 900 to 1900 nm and the incident angle is below 39 degrees. As we see, the is a slight variation of absorption spectra under oblique incidence, particularly for p-polarized (TM) light where the resonance at the short wavelength splits at large incident angles. However, for both s-polarized (TE) and p-polarized (TM) light, the light absorption by monolayer graphene keeps higher than 27% in a spectral range from 900 to 1760 nm for incident angle up to 39 degrees.

## Conclusions

In summary, we have numerically investigated three different plasmonic absorber designs for light trapping and absorption enhancement in graphene. The excitation of localized plasmons in the metallic structures can significantly enhance the interactions between graphene and light at the resonances. By employing a splitted cross design for plasmonic resonant antennas, dual resonances can be obtained in the studied spectral range. Furthermore, by integrating two types of sub-antennas with different sizes, the bandwidth for light trapping and absorption enhancement is effectively expanded due to multi-resonances. More than one order enhancement of optical absorption in a single layer graphene is realized in an unprecedented broad spectral range from 780 to 1760 nm. This enhancement functionality can be translated to any wavelength range from visible to terahertz by modifying the geometric design of the plasmonic structure and can be applied for other two dimensional materials and their heterogeneous structures. Besides, the proposed design is compact and suitable for integrated thin film devices which is an advantage over some other proposed methods for absorption enhancement in graphene^46^,^47^. The proposed metallic nanostructures can be fabricated by e-beam lithography, nano-imprint or other nano-fabrication techniques^48^,^49^. The designs may significantly improve the efficiency of optical devices such as broadband photodetectors and solar cells based on graphene and other two-dimensional materials.

## Methods

The numerical simulations are conducted using a fully three-dimensional finite element technique (in Comsol MultiPhysics). In the simulation, the graphene is modelled as a conductive surface with an optical conductance of *G*_0_ = *e*^2^/(4*ħ*) ≈ 6.08 × 10^−5^ Ω^−1^ which corresponds to the absorption of about 2.3% for a free standing film. The permittivity of gold was described by the Drude model with plasma frequency *ω*_*p*_ = 1.37 × 10^16^ *s*^−1^ and the damping constant *ω*_*τ*_ = 1.23 × 10^14^ *s*^−1^ which was three times larger than the bulk value considering the increased scattering by surface and grain boundary effects in the thin film.

## Additional Information

**How to cite this article**: Xiong, F. *et al.* Ultrabroadband, More than One Order Absorption Enhancement in Graphene with Plasmonic Light Trapping. *Sci. Rep.* **5**, 16998; doi: 10.1038/srep16998 (2015).
